# Clinical Outcomes of Poly(ADP–Ribose) Polymerase Inhibitors as Maintenance Therapy in Patients with Ovarian Cancer in the Southeastern Region of Korea

**DOI:** 10.3390/curroncol31110495

**Published:** 2024-10-28

**Authors:** Hyeong In Ha, Hyung Joon Yoon, Changho Song, Eun Taeg Kim, Dong-Soo Suh, Ki Hyung Kim, Yong Jin Na, Yong Jung Song

**Affiliations:** 1 Department of Obstetrics and Gynecology, Pusan National University College of Medicine, Busan 49241, Republic of Korea; hi126908111@gmail.com (H.I.H.); princeps21@naver.com (H.J.Y.); dssuh@pusan.ac.kr (D.-S.S.); ghkim@pusan.ac.kr (K.H.K.); yjna@pusan.ac.kr (Y.J.N.); 2 Department of Obstetrics and Gynecology, Pusan National University Yangsan Hospital, Yangsan-si 50612, Republic of Korea; 3 Biomedical Research Institute, Pusan National University Hospital, Busan 49241, Republic of Korea; 4 Department of Obstetrics and Gynecology, Ulsan University Hospital, University of Ulsan College of Medicine, Ulsan 44033, Republic of Korea; 0736355@uuh.ulsan.kr; 5 Department of Obstetrics and Gynecology, Kosin University Gospel Hospital, Kosin University College of Medicine, Busan 49267, Republic of Korea; dikei03@naver.com

**Keywords:** ovarian cancer, PARP inhibitor

## Abstract

Purpose: In this study, we aimed to retrospectively investigate the real-world clinical efficacy and adverse events of poly(adenosine diphosphate [ADP]-ribose) polymerase (PARP) inhibitors in real-world clinical practice among patients with newly diagnosed epithelial ovarian cancer. Methods: We retrospectively reviewed the medical records from hospitals. Patients with epithelial ovarian cancer treated with olaparib or niraparib as frontline maintenance treatment between 1 January 2014 and 31 December 2022 were included. Progression-free survival (PFS) was analyzed using the Kaplan–Meier method, and adverse events associated with PARP inhibitor treatment were investigated. Results: Ninety-six patients treated with PARP inhibitors were identified. The median follow-up period was 21.8 months (95% confidence interval [CI] 19.4–24.0). Twenty (20.1%) patients experienced disease progression, and two patients died. The median PFS was 45.3 months (95% CI 39.4–NA). *BRCA1* or *BRCA2* gene mutations and primary cytoreductive surgery were associated with better PFS. Adverse events of any grade occurred in 74 (77.1%) patients. Nineteen (19.8%) patients experienced PARP inhibitor therapy interruptions, and 35 (36.5%) patients experienced dose reductions. Only three patients discontinued the drug due to adverse events. Conclusions: In a real-world setting, PARP inhibitors showed efficacy comparable to that reported in published randomized controlled trials and had acceptable safety profiles.

## 1. Introduction

Cancer has been a leading cause of death in Korea in recent years. Ovarian cancer, which includes fallopian tube and primary peritoneal cancers, is the second most common type of gynecological cancer [1]. It is the leading cause death from gynecological malignancies. Despite its lower incidence compared to uterine cancer, ovarian cancer has a higher mortality rate. It is often diagnosed at an advanced stage and is prone to recurrence, with a significant proportion of patients diagnosed at FIGO stage III or IV. According to data from the Korea Central Cancer Registry, which has collected information on approximately 98% of all cancer cases in Korea since 1999 [2], only 28% of patients with ovarian cancer are in the localized Surveillance, Epidemiology, and End Results (SEER) stage at diagnosis, while 17.4% are in the Regional stage, and 45.4% are in the Distant stage [3].

The optimal treatment for advanced ovarian cancer is primary debulking surgery as a first step to minimize the tumor burden, followed by platinum-based chemotherapy. On the other hand, for patients with certain conditions such as poor performance status, old age, unresectable metastatic tumors and medical comorbidities, neoadjuvant chemotherapy is considered first followed by interval debulking surgery [4]. However, despite these treatments, the recurrence rate of ovarian cancer is high, and more than 70% of stage III-IV high-grade ovarian cancer patients experience recurrence within 3 years [5]. Recurrent ovarian cancer is difficult to treat because it often shows molecular alteration of original tumors and resistance to anticancer drugs. Therefore, the need to provide appropriate maintenance treatment to prevent disease recurrence has been proposed. The goal of maintenance therapy is to prolong the benefits of chemotherapy and improve progression-free and overall survival without compromising patient quality of life or increasing toxicity [6].

One of the representative drugs used for the maintenance treatment of advanced newly diagnosed ovarian cancer is bevacizumab, a humanized vascular endothelial growth factor (VEGF)-neutralizing monoclonal antibody. The drug had progression-free survival (PFS) benefits in the multicenter, randomized, double-blind, placebo-controlled study GOG-0218 (NCT00262847) [7]. However, the final results of this study showed no improvement in overall survival [8]. Additionally, because bevacizumab is administered intravenously, it has the inconvenience of requiring patients to visit the hospital every three weeks.

Another class of maintenance agents for newly diagnosed ovarian cancer includes poly (adenosine diphosphate [ADP]-ribose) polymerase (PARP) inhibitors, such as olaparib and niraparib. Both have demonstrated efficacy in treating primary and recurrent epithelial ovarian cancer in randomized controlled trials (RCTs) [9,10,11,12,13]. However, real-world clinical outcomes may differ from those observed in RCTs owing to various factors. Therefore, additional studies involving real-world patients are needed. In this study, we aimed to retrospectively investigate the clinical efficacy and adverse events of PARP inhibitors in real-world clinical practice among patients with newly diagnosed epithelial ovarian cancer.

## 2. Materials and Methods

This retrospective multicenter study included patients from four Korean institutions across Southeast Gyeongsang Province, Korea: Pusan National University Hospital, Pusan National University Yangsan Hospital, Ulsan University Hospital, and Kosin University Hospital. Through the retrospective chart review, clinical data such as demographic, clinicopathological, and surgical records for this study were collected. Participants had pathologically diagnosed epithelial ovarian cancer, fallopian tube cancer, or primary peritoneal cancer and underwent primary cytoreductive surgery or interval debulking surgery after neoadjuvant chemotherapy followed by platinum-based chemotherapy as frontline treatment. Included patients showed complete or partial response to at least four cycles of platinum-based frontline chemotherapy and were treated with Poly(ADP–Ribose) Polymerase inhibitors as maintenance therapy either olaparib or niraparib between 1 January 2014 and 31 December 2022. The patients with insufficient clinicopathological data were excluded. All patients underwent BRCA testing to identify germline or somatic pathogenic/likely pathogenic variants of BRCA1 or BRCA2. Tests for germline BRCA1 or BRCA2, Sanger sequencing, next-generation sequencing (NGS), or NGS-based somatic BRCA1 or BRCA2 were performed.

The clinical outcome was measured in the form of PFS. PFS was defined as the interval from the end of platinum-based adjuvant chemotherapy to disease progression according to the Response Evaluation Criteria in Solid Tumors (RECIST) 1.1, or death from any cause, or last follow-up date. To find evidence of recurrence, regularly performed radiological assessments were also reviewed and CA125 level was checked. The follow-up interval was every 3 months for up to 3 years after the completion of first-line chemotherapy, and subsequently every 6 months for up to 5 years. The radiological assessments include abdominal and pelvic computed tomography (CT), chest CT. Magnetic resonance imaging (MRI), or positron emission tomography-computed tomography (PET-CT) can be used for additional information. Adverse events occurring during PARP inhibitor treatment were documented and graded according to the National Cancer Institute Common Terminology Criteria for Adverse Events (CTCAE) version 5.0. The dose interruption or reduction of PARP inhibitors due to adverse events was also documented. Laboratory test results, including complete blood count, chemistry profile were reviewed to assess and grade the severity of adverse events and any specific medico-surgical events were documented.

For categorical variables, patient characteristics were presented as frequencies and percentages. For continuous variables that followed normal distribution, the characteristics were presented as medians and ranges. Survival outcome was analyzed using the Kaplan–Meier method, and differences in cumulative survival were assessed using the log-rank test. Patients who did not experience an event by the last follow-up on 31 December 2022, were censored. Univariate analysis was performed to identify factors associated with longer PFS. Variables with a significance level of *p* < 0.05 in the univariate analysis were included in the multivariate Cox regression analysis. All statistical analyses were performed using R Project 4.3.1 (R Foundation for Statistical Computing, Vienna, Austria)

The study protocol was reviewed and approved by the Institutional Review Board of Pusan National University Yangsan Hospital (No. 55-2024-006). The requirement for informed consent was waived due to the secondary analysis of de-identified data.

## 3. Results

Ninety-six patients treated with PARP inhibitors were identified and included in this study. Patient characteristics are summarized in Table 1. The median age at diagnosis was 54 years, and the median body mass index was 23.0 kg/m^2^ (range: 16.7–40.5). All patients underwent cytoreductive surgery regardless of neoadjuvant chemotherapy. Seventy-seven patients (80.2%) underwent primary cytoreductive surgery, while 19 patients (19.8%) received paclitaxel plus carboplatin neoadjuvant chemotherapy followed by interval cytoreductive surgery. All patients received chemotherapy with a paclitaxel plus carboplatin regimen, except for one with paclitaxel hypersensitivity who received docetaxel plus carboplatin.

None of the patients exhibited disease progression after adjuvant chemotherapy based on the RECIST 1.1 criteria. Thirty-three patients (34.4%) exhibited a partial response, and 62 patients (64.6%) showed a complete response to adjuvant chemotherapy. The median CA-125 levels at diagnosis were 1069 U/mL (range: 22.5–12,920) for patients who received neoadjuvant chemotherapy and 604.5 U/mL (range: 11.9–10,630) for those who underwent primary debulking surgery. At the time of cytoreductive surgery, 59 patients had CA-125 levels < 35 U/mL. Following adjuvant chemotherapy and PARP inhibitor treatment, 88 patients (91.6%) had normalized CA-125 levels.

Patients were treated with olaparib or niraparib. Twenty-seven patients (28.1%) received olaparib (300 (table) or 400 mg (capsule) twice daily), and 64 patients (66.7%) received niraparib (200 mg once daily).

To assess genetic predisposition to epithelial ovarian, primary peritoneal, and fallopian tube cancers, all patients were evaluated for BRCA1 and BRCA2 gene mutations. Fifty-eight patients (60.4%) had wild-type genes, while 19 patients (19.8%) had pathogenic or likely pathogenic variants in BRCA1 and another 19 patients (19.8%) in BRCA2. All patients treated with olaparib had BRCA1 or BRCA2 mutations. Among those treated with niraparib, 58 had wild-type genes and 11 had pathogenic or likely pathogenic variants of BRCA1 or BRCA2 (Supplementary Table S1).

Disease characteristics are described in Table 2. Most patients had epithelial ovarian cancer (*n* = 91, 94.8%), with three (3.1%) having fallopian tube cancer and two (2.1%) having primary peritoneal cancer. Regarding tumor histology, 82 patients (85.4%) had high-grade serous carcinoma, and seven (7.3%) had endometrioid histology. Additionally, patients with low-grade serous carcinoma (*n* = 2, 2.1%) and undifferentiated large-cell neuroendocrine clear-cell carcinoma were included. Eighty-three patients (86.4%) had advanced FIGO stages, while nine (9.4%) were FIGO stage I, and four (4.2%) were FIGO stage II. After cytoreductive surgery, 65 patients (67.7%) had no residual cancer, and 31 (32.3%) had residual tumors with a long axis of <1 cm in size. Only 12 patients (12.5%) had residual tumors with a long axis > 1 cm.

**Figure 1:**
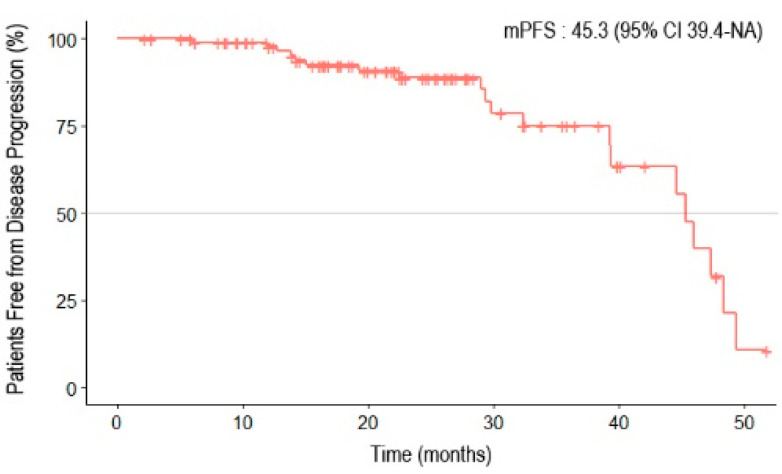
Progression-free survival of patients with epithelial ovarian, primary peritoneal, and fallopian tube cancers receiving first-line PARP inhibitor maintenance treatment.

**Figure 2:**
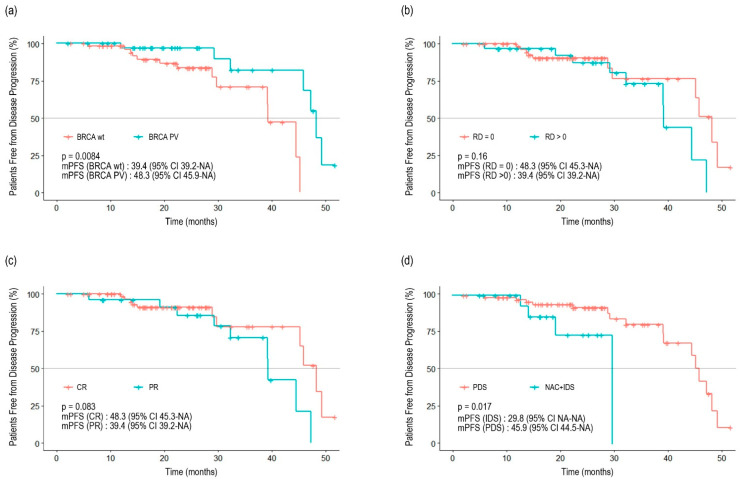
Progression-free survival stratified by *BRCA1* and *BRCA2* mutation status (**a**), residual disease (**b**), previous response to adjuvant chemotherapy (**c**), and type of surgery (**d**).

The median follow-up period was 21.8 months (95% confidence interval [CI] 19.4–24.0). The median duration of administration was 13.6 months (range: 0.2–44.8) for olaparib and 18.2 months (range: 1–38.4) for niraparib. Twenty patients (20.1%) experienced disease progression, and two patients died. The Kaplan–Meier survival curve for PFS is shown in Figure 1. The median PFS (mPFS) was 45.3 months (95% CI 39.4–NA). In the subgroup analysis, no differences were observed between the Kaplan–Meier curves based on residual disease or chemotherapy response (Figure 2). The results from the univariate analysis indicated that BRCA mutations and primary cytoreductive surgery were associated with better PFS. However, multivariate analysis revealed that BRCA1 or BRCA2 mutations were significantly associated with better prognosis (*p* = 0.025) (Table 3).

Seventy-five patients (78.1%) continued PARP inhibitor treatment, while 21 patients (21.8%) discontinued it. Reasons for discontinuation included disease progression (15 patients), adverse events (three patients), and insurance issues (three patients). Additionally, 19 patients (19.8%) experienced temporary delays in PARP inhibitor administration, and 35 (36.5%) required dose reductions (Table 4).

Adverse events of any grade occurred in 74 patients (77.1%). Anemia was the most common adverse event, affecting 57 patients (59.4%), with eight (8.3%) experiencing CTCAE grade 3 or 4 anemia. Other common adverse events included neutropenia and thrombocytopenia. Non-hematologic adverse events, such as gastrointestinal symptoms (e.g., nausea and vomiting), fatigue, abdominal pain, and constipation, were mostly CTCAE grade 1 or 2. No secondary malignancies, such as acute myeloid leukemia or myelodysplastic syndromes, were reported in this study (Table 5).

## 4. Discussion

PARP is a family of 17 nucleoproteins [14] crucial for DNA damage repair. This nuclear enzyme catalyzes the transfer of ADP-ribose residues from nicotinamide adenine dinucleotide onto target substrates to build poly(ADP-ribose) (PAR) chains [15]. Human cells constantly undergo DNA damage, which can be categorized into single-strand breaks (SSBs) and double-strand breaks (DSBs). PARP plays a role in repairing SSBs through a pathway known as base excision repair (BER). When PARP functions normally, SSBs are effectively repaired via BER. However, compromised PARP function or the use of PARP inhibitors prevents SSB repair. During DNA replication, an SSB can transform into a DSB. While normal cells can repair DSBs through homologous recombination (HR), HR-deficient tumor cells cannot repair the damage and undergo apoptosis. This mechanism underscores the effectiveness of PARP inhibitors, making them particularly effective in patients with BRCA mutations, which are a key cause of HR deficiency [15,16].

PARP inhibitors used for ovarian cancer treatment include olaparib, niraparib, and rucaparib, with olaparib and niraparib being the most used in Korea. Olaparib primarily inhibits PARP1 and PARP2 [17], while niraparib exhibits potent and selective inhibition of PARP1 and PARP2, with activity against these enzymes being 100-fold higher than against other PARP family members (PARP3, v-PARP and Tankyrase-1) [18]. Each drug’s efficacy has been demonstrated in landmark RCTs. The SOLO1 trial (NCT01844986) is pivotal for the maintenance treatment of newly diagnosed epithelial ovarian cancer. The study included patients with advanced (FIGO stage III or IV) epithelial ovarian cancer and BRCA1 and/or BRCA2 mutations. Among 391 patients with a median follow-up of 41 months, the risk of death or disease progression was 70% lower with olaparib compared to placebo (hazard ratio for disease progression or death, 0.30; 95% CI 0.23–0.41) [10]. At the time of publication, the median PFS of the study was not reached. Three years later, Banerjee et al. reported the 5-year follow-up data and PFS of the SOLO1 study [19]. The median PFS was 56.0 months (41.9–NR) and the hazard ratio was 0.33 (95% Cl 0.25–0.43). Despite its efficacy, the SOLO1 study focused exclusively on patients with BRCA1/2 mutations, which are present in only 5–15% of ovarian cancer cases [20]. This has increased interest in exploring maintenance treatments for patients with BRCA1 or BRCA2 wild-type status.

Conversely, the PRIMA study (NCT02655016) expanded its inclusion criteria to encompass not only patients with BRCA1 or BRCA2 mutations but also those with HR proficiency [13]. This study included 733 patients, of whom 373 (50.9%) had tumors with HR deficiency. In the PRIMA study, PFS was 13.8 months in the niraparib group compared to 8.2 months in the placebo group (hazard ratio, 0.62; 95% CI, 0.50–0.76; *p* < 0.001). The study only included patients with stage III disease with visible residual tumor after primary debulking surgery, inoperable stage III disease, or any stage IV disease. The presence of residual disease may influence clinical outcomes and explain the differences observed in the median PFS between the PRIMA study and this retrospective study. Thus, to accurately assess the efficacy of niraparib, another randomized controlled trial named the PRIME study was implemented [21]. This study included patients who underwent primary or interval debulking surgery, regardless of residual disease. The median PFS of the PRIME study was 24.8 months (19.2–NA) longer than that in the PRIMA study.

PARP inhibitors have demonstrated efficacy not only as first-line maintenance therapy but also as recurrent ovarian cancer maintenance therapy. The SOLO2 trial evaluated olaparib maintenance therapy in patients with platinum-sensitive BRCA1 or BRCA2 mutations and relapsed ovarian cancer. Olaparib significantly improved PFS compared to placebo (19.1 vs. 5.5 months; HR 0.3; *p* < 0.0001) [11]. Although the SOLO2 study confirmed a high effectiveness, it was limited to patients with BRCA1 or BRCA2 mutations.

Conversely, the NOVA trial (NCT01847274) assessed the clinical effectiveness of niraparib regardless of BRCA status. In the overall non-germline BRCA (non-gBRCA) cohort, patients treated with niraparib had a longer median PFS than those in the placebo group (9.3 months vs. 3.9 months; hazard ratio, 0.45; 95% CI, 0.34–0.61) [12]. These phase III RCTs consistently reported significant improvements, providing robust evidence supporting the use of PARP inhibitors in ovarian cancer maintenance therapy.

In the present study, treatment-emergent adverse events occurred in 74 (77.1%) patients, with 16 (16.7%) experiencing grade 3 or 4 adverse events according to CTCAE criteria. In the SOLO1 study, adverse events of any kind were observed in 98% of patients, with nausea being the most common [10,22]. Similarly, in the PRIMA study, which evaluated niraparib maintenance treatment, approximately 98.8% of patients experienced adverse events, with anemia (63.4%) being the most common, followed by nausea (57.4%) [13,23]. In the present study, the most common adverse event was neutropenia (64.6%), followed by anemia (59.4%). There were no reports of hematological malignancies, such as acute myeloid leukemia or myelodysplastic syndrome. Only 13.5% of patients reported nausea or vomiting. Overall, the incidence of adverse events in the present study was relatively low compared to that of RCTs. Since this study was retrospective, patient-reported adverse events such as nausea and fatigue were not systematically recorded and may be underestimated. Additionally, minor adverse effects of grades 1 and 2 may not have been reported by patients to their healthcare providers. Moreover, unlike RCTs that included patients with advanced-stage ovarian cancer, this study included patients with FIGO stages I and II (13.5%). Furthermore, while the RCT protocol initially prescribed a fixed dose for all patients, the concept of an individualized starting dose was incorporated midway through the trial. Conversely, the patients in the present study were administered PARP inhibitors based on an individualized starting dose protocol (starting with 300 mg once daily for patients weighing ≥ 77 kg and with platelets ≥ 150,000/μL, otherwise 200 mg once daily). Therefore, while more rigorous prospective recordings of adverse events might reveal a higher incidence rate, it appears that most patients can tolerate PARP inhibitors relatively safely.

National policies on insurance reimbursement for PARP inhibitors significantly impact treatment in Korea owing to their high cost. In October 2019, the Ministry of Food and Drug Safety of Korea approved olaparib as a first-line maintenance treatment for patients newly diagnosed with advanced, platinum-sensitive epithelial ovarian cancer with BRCA1 or BRCA2 pathogenic or likely pathogenic variants. Furthermore, in October 2021, the National Health Insurance Service of Korea began to reimburse first-line olaparib maintenance treatment for up to 2 years after the first dose. Similarly, in September 2020, the Ministry approved niraparib as a maintenance treatment for patients with newly diagnosed, platinum-sensitive epithelial ovarian cancer, regardless of BRCA1 or BRCA2 status. Niraparib has been reimbursed by the National Health Insurance Service since October 2021 for patients with BRCA1 or BRCA2 pathogenic or likely pathogenic variants. In the present study, three patients discontinued PARP inhibitor treatment owing to financial concerns. Two of these patients had FIGO stage I disease, which is not considered advanced, and the other patient could not afford the treatment without National Health Insurance Service coverage. All patients who used olaparib had BRCA1 or BRCA2 gene mutations. This is because olaparib was not approved in Korea for use in patients without BRCA mutations. Among those using niraparib, the majority (84.1%) had wild-type BRCA1 or BRCA2 genes, and niraparib was approved regardless of BRCA1 or BRCA2 status. Similarly, Shin et al. reported the results of 32 patients using niraparib from July 2020 to January 2021 in a study analyzing the usage pattern of niraparib [24]. Among these patients, 26 (83%) had wild-type BRCA1 or BRCA2 genes. Additionally, Chen et al. reported that among 60 Chinese patients who used niraparib as first-line maintenance treatment, 86.7% had wild-type genes [25].

Generally, patients with residual tumors after cytoreductive surgery have a worse prognosis than those without residual cancer [26,27]. However, in the present study, the Kaplan–Meier curves intersected, indicating no statistically significant difference between the two groups (*p* = 0.16). Unlike RCTs that enforce strict inclusion criteria, such as including only advanced-stage patients and requiring an Eastern Cooperative Oncology Group performance status of 0 or 1, the present study included patients at any disease stage and with varying general conditions. Furthermore, owing to health insurance policies, olaparib administration in Korea is limited to 24 months under insurance reimbursement, which has led some patients to discontinue PARP inhibitor treatment for non-medical reasons. However, these differences should be carefully interpreted, they reflect the practices observed in the southeastern region of Korea rather than being representative of the entire country.

This study presents the real-world clinical outcomes and adverse events of first-line PARP inhibitor maintenance treatment for patients with epithelial ovarian cancer in the Southeast area of Korea. Unlike recurrent ovarian cancer, there are limited reports on first-line PARP inhibitor maintenance therapy for epithelial ovarian cancer. As more data accumulates, the efficacy of PARP inhibitors in actual clinical trials will be more rigorously validated.

This study had several limitations such as a small sample size, relatively unrepresentative cohort, short follow-up period, and underestimated adverse events owing to its retrospective nature. Thus, additional real-world data collection is crucial to accurately determine the effectiveness of PARP inhibitors in clinical settings. Furthermore, with the introduction of various combinations of medications, such as VEGF inhibitors, programmed death-ligand 1 inhibitors, and PARP inhibitors in ovarian cancer treatment, systematic and diverse clinical trials are necessary to establish optimal treatment algorithms for patients with ovarian cancer.

In conclusion, the results from this study indicate that PARP inhibitors demonstrate effectiveness comparable to that reported in RCTs and maintain an acceptable safety profile in a real-world setting.

## Figures and Tables

**Table 1:** Patients characteristics.

Characteristics	*n* (Total = 96) (%)
Age (yr) (median, range)	54 (32–82)
Height (cm) (mean)	157.0
Weight (kg) (mean)	55.8
<77 kg	95 (98.9)
≥77 kg	1 (1.1)
BMI (median, range)	23.0 (16.7–40.5)
Type of surgery	
Primary debulking surgery	77 (80.2)
Interval debulking surgery	19 (19.8)
Type of adjuanvt chemotherapy preceding PARPi	
Paclitaxel + Carboplatin	85 (88.5)
Paclitaxel + Carboplatin + Bevacizumab	10 (10.4)
Docetaxel + Carboplatin	1 (1.0)
CA 125 level at the time of diagnosis (median) U/mL	
Patients with primary debulking surgery	604.5 (11.9–10,630)
Patients with neoadjuvant chemotherapy	1069 (22.5–12,920)
CA 125 level after adjuvant chemotherapy, before PARP inhibitor use	
<35 U/mL	88 (91.6)
≥35 U/mL	4 (4.2)
NA	4 (4.2)
Type of PARP inhibitor	
Olaparib	27 (28.1)
Niraparib	69 (71.8)
Starting dose	
Olaparib 300 mg bid (tablet)	23 (23.9)
Olaparib 400 mg bid (capsule)	4 (4.2)
Niraparib 100 mg Qd	4 (4.2)
Niraparib 200 mg Qd	64 (66.7)
Niraparib 300 mg Qd	1 (1.0)
*BRCA1* and *BRCA2* status	
Wild-type	58 (60.4)
*BRCA1* mutated	19 (19.8)
*BRCA2* mutated	19 (19.8)

**Table 2:** Disease characteristics.

Characteristics	*n* (Total = 96) (%)
Tumor origin	
Epithelial ovarian cancer	91 (94.8)
Fallopian tube	3 (3.1)
Primary peritoneal cancer	2 (2.1)
Tumor histology	
High-grade serous carcinoma	82 (85.4)
Endometrioid carcinoma	7 (7.3)
Others	5 (5.2)
FIGO stage	
I	9 (9.4)
II	4 (4.2)
III	58 (60.4)
IV	25 (26.0)
Residual tumor size	
Microscopic	65 (67.7)
≤1 cm	19 (19.8)
>1 cm	12 (12.5)
Response of adjuvant chemotherapy	
Complete response	62 (64.6)
Partial response	33 (34.4)
Stable Disease	1 (1.0)

**Table 3:** Univariate and multivariate analysis.

	Progression-Free Survival (Event = 20)			
	Univariate		Multivariate	
	HR (95% CI)	*p*-Value	HR (95% CI)	*p*-Value
*BRCA1* or *BRCA2* status				0.025
Wild-type	1		1	
*BRCA1* or *BRCA2* PV	0.2 (0.05–0.73)	0.008	0.22 (0.06–0.83)	
Residual disease				
absent	1			
present	1.96 (0.76–5.09)	0.16		
response to chemotherapy				
complete response	1			
partial response	2.27 (0.88–5.87)	0.083		
Type of surgery				0.070
PDS	1		1	
NAC + IDS	4.23 (1.17–15.3)	0.017	3.23 (0.91–11.5)	

HR = Hazard Ratio, CI = Confidence interval, PDS = primary debulking surgery, NAC = neoadjuvant chemotherapy, IDS = interval debulking surgery, PV = pathogenic variant.

**Table 4:** Dose change for adverse events during PARP inhibitor use.

	Total (*n* = 96)	Olaparib (*n* = 27)	Niraparib (*n* = 69)	*p*-Value
duration of use (mo)	16.8	14.6	17.6	0.17
dose interruption	19 (19.8%)	8 (29.6%)	11 (15.9%)	0.13
dose reduction	35 (36.5%)	9 (33.3%)	26 (37.7%)	0.691
discontinuation	21 (21.9%)	9 (33.3%)	12 (17.4%)	0.089
due to side effect	3 (3.1%)	1 (3.7%)	2 (2.9%)	0.838
due to progression	15 (15.6%)	6 (22.2%)	9 (13.0%)	0.265
due to non-medical reason	3 (3.1%)	2 (7.4%)	1 (1.4%)	0.09

**Table 5:** Common adverse events during PARP inhibitor use.

	Total (*n* = 96)	Olaparib (*n* = 27)	Niraparib (*n* = 69)	*p*-Value
Any adverse events				
any grade	74 (77.1%)	22 (81.5%)	52 (75.4%)	0.521
grade ≥ 3	16 (16.7%)	4 (14.8%)	12 (17.4%)	0.761
Anemia				
any grade	57 (59.4%)	16 (59.3%)	41 (59.4%)	0.988
grade ≥ 3	8 (8.3%)	3 (11.1%)	5 (7.2%)	0.538
Thrombocytopenia				
any grade	19 (19.8%)	4 (14.8%)	15 (21.7%)	0.444
grade ≥ 3	8 (8.3%)	1 (3.7%)	7 (10.1%)	0.305
neutropenia				
any grade	62 (64.6%)	21 (77.8%)	41 (59.4%)	0.091
grade ≥ 3	5 (5.2%)	2 (7.4%)	3 (4.3%)	0.544
MDS/AML				
any grade	0 (0.0%)	0 (0.0%)	0 (0.0%)	NA
grade ≥ 3	0 (0.0%)	0 (0.0%)	0 (0.0%)	NA
Nausea/Vomiting				
any grade	13 (13.5%)	6 (22.2%)	7 (10.1%)	0.12
grade ≥ 3	0 (0.0%)	0 (0.0%)	0 (0.0%)	NA
Constipation				
any grade	2 (2.1%)	0 (0.0%)	2 (2.9%)	0.371
grade ≥ 3	0 (0.0%)	0 (0.0%)	0 (0.0%)	NA
Abdominal pain				
any grade	1 (1.0%)	0 (0.0%)	1 (1.4%)	0.529
grade ≥ 3	0 (0.0%)	0 (0.0%)	0 (0.0%)	NA
Fatigue				
any grade	4 (4.2%)	2 (7.4%)	2 (2.9%)	0.320
grade ≥ 3	0 (0.0%)	0 (0.0%)	0 (0.0%)	NA
Dizziness				
any grade	2 (2.1%)	1 (3.7%)	1 (1.4%)	0.487
grade ≥ 3	0 (0.0%)	0 (0.0%)	0 (0.0%)	NA
Others				
any grade	8 (8.3%)	0 (0.0%)	8 (11.6%)	
grade ≥ 3	3 (3.1%)	0 (0.0%)	3 (4.3%)	

## Data Availability

The original contributions presented in the study are included in the article/Supplementary Materials, further inquiries can be directed to the corresponding author.

## Supplementary Materials

The following supporting information can be downloaded at: https://www.mdpi.com/article/10.3390/curroncol31110495/s1, Table S1: Patients Characteristics according to PARP inhibitor type; Table S2: Toxicities of poly (ADP-ribose) polymerase (PARP) inhibitors described in the phase 3 trial.
