# Supplementary material for: Clinical Outcomes of Poly(ADP–Ribose) Polymerase Inhibitors as Maintenance Therapy in Patients with Ovarian Cancer in the Southeastern Region of Korea
Source: Curr Oncol. 2024 Oct 28;31(11):6711–22. doi: 10.3390/curroncol31110495 (PMC11593207; doi:10.3390/curroncol31110495)
Supplement: Supplementary file 1 [file curroncol-31-00495-s001.zip › curroncol-3246581-supplementary.pdf]

Supplementary Table S1. Patients Characteristics according to PARP inhibitor type.

Characteristics	Olaparib (n = 27)	Niraparib (n=69)	p-value
Age (yr) (median, range)	57 (45-82)	52 (32-76)	0.891
Height (cm) (mean)	156.3	157.3	0.734
Weight (kg) (mean)	53.6	57.8	0.325
BMI (median)	21.9	23.7	0.216
Duration of use (mean,range)	13.6 (0.2 – 44.8)	17.7 (1.0 – 38.4)	0.325
Type of surgery			0.774
Primary debulking surgery	22	55	
Interval debulking surgery	5	14	
BRCA1 and *BRCA2* status			<0.001
wild type	0	58	
BRCA1 mutated	15	4	
BRCA2 mutated	12	7	

Supplementary Table S2. Toxicities of poly (ADP-ribose) polymerase (PARP) inhibitors described in the phase 3 trial.

	PRIMA-Niraparib (n=484)		PRIMA-Placebo (n=244)		SOLO1- Olaparib (n=260)		SOLO1-Placebo (n=130)		Present study (n=96)	
	All grades	Grade 3 or 4	All grades	Grade 3 or 4	All grades	Grade 3 or 4	All grades	Grade 3 or 4	All grades	Grade 3 or 4
Anemia	307 (63.4%)	150 (31.0%)	43 (17.3%)	4 (1.6%)	101 (39%)	56 (22%)	13 (10%)	2 (2%)	57 (59.4%)	8 (8.3%)
Thrombocytope-nia	222 (45.9%)	139 (28.1%)	9 (3.7%)	1 (0.4%)	29 (11%)	2 (1%)	5 (4%)	2 (2%)	19 (19.8%)	8 (8.3%)
Neutropenia	128 (26.4%)	62 (12.8%)	16 (6.6%)	3 (1.2%)	60 (23%)	22 (9%)	15 (12%)	6 (5%)	62 (64.6%)	5 (5.2%)
Nausea	278 (57.4%)	6 (1.2%)	67 (27.5%)	2 (0.8%)	201 (77%)	2 (1%)	49 (38%)	0 (0%)	13 (13.5%)	0 (0%)
Constipation	189 (39.0%)	1 (0.2%)	46 (18.9%)	0 (0%)	72 (28%)	0 (0%)	25 (19%)	0 (0%)	2 (2.1%)	0 (0%)
Vomiting	108 (22.3%)	4 (0.8%)	29 (11.9%)	2 (%)	104 (40%)	1(<1%)	19 (15%)	1 (<1%)	13 (13.5%)	0 (0%)
Decreased appe-tite	NA	NA	NA	NA	51 (20%)	0 (0%)	13 (10%)	0 (0%)	NA	NA
Abdominal pain	106 (21.9%)	7 (1.4%)	75 (30.7%)	1 (0.4%)	64 (25%)	4 (2%)	25 (19%)	1 (<1%)	1 (1.0%)	0 (0%)
Diarrhea	NA	NA	NA	NA	89 (34%)	8 (3%)	32 (25%)	0 (0%)	NA	NA
Dyspepsia	NA	NA	NA	NA	43 (17%)	0 (0%)	16 (12%)	0 (0%)	NA	NA
Dysgeusia	NA	NA	NA	NA	68 (26%)	0 (0%)	5 (4%)	0 (0%)	NA	NA
Fatigue	168 (34.7%)	9 (1.9%)	72 (29.5%)	1 (0.4%)	165 (63%)	10 (4%)	54 (42%)	2 (2%)	4 (4.2%)	0 (0%)
Dizziness	NA	NA	NA	NA	51 (20%)	0 (0%)	20 (15%)	1 (<1%)	2 (2.1%)	0 (0%)
Headache	126 (26.0%)	2 (0.4%)	36 (14.8%)	0 (0%)	59 (23%)	1(<1%)	31 (24%)	3 (2%)	NA	NA
Dyspnea	NA	NA	NA	NA	39 (15%)	0 (0%)	7 (5%)	0 (0%)	NA	NA
Nasopharyngitis	NA	NA	NA	NA	NA	NA	NA	NA	NA	NA
Cough	NA	NA	NA	NA	42 (16%)	0 (0%)	28 (22%)	0 (0%)	NA	NA
Arthralgia	NA	NA	NA	NA	66 (25%)	0 (0%)	35 (27%)	0 (0%)	NA	NA
